# Plastic Smell: A Review of the Hidden Threat of Airborne Micro and Nanoplastics to Human Health and the Environment

**DOI:** 10.3390/toxics13050387

**Published:** 2025-05-12

**Authors:** Claudio Casella, Umberto Cornelli, Santiago Ballaz, Giuseppe Zanoni, Gabriele Merlo, Luis Ramos-Guerrero

**Affiliations:** 1 Department of Chemistry, University of Pavia, Viale Taramelli 12, 27100 Pavia, Italy; icarocus@gmail.com (C.C.); gz@unipv.it (G.Z.); gabriele.merlo02@universitadipavia.it (G.M.); 2 School of Medicine, Loyola University, Chicago, IL 60660, USA; ucornelli@gmail.com; 3 Faculty of Health Sciences, Universidad del Espiritu Santo, Samborondón P.O. Box 09-01-952, Ecuador; sballazg@gmail.com; 4 Grupo de Investigación en Bio-Quimioinformática, Carrera de Ingeniería Agroindustrial, Facultad de Ingeniería y Ciencias Aplicadas, Universidad de Las Américas (UDLA), Quito 170513, Ecuador

**Keywords:** airborne microplastics and nanoplastics, human health, sampling methods, indoor and outdoor, dust

## Abstract

Airborne micro and nanoplastics (MPs/NPs) are a growing issue due to their possible health hazards. Since the current bibliography lacks a thorough evaluation, this review examines the sources, environmental dynamics, and health impacts of airborne MPs/NPs. Through atmospheric transport processes, these neo-pollutants spread around the world after being released, potentially settling in urban and remote areas. This review is the first to compare active and passive aerosol sampling methods, and microscopy, thermochemical, and spectroscopy analytical techniques, with a focus on their limitations in precisely quantifying micro-nanoscale plastic particles. It also draws attention to the potential toxicological effects of inhaled MPs/NPs, which can lead to oxidative stress, respiratory inflammation, and other negative health consequences. This review concludes by examining how airborne MPs/NPs may worsen their ecological impact by serving as carriers of hazardous chemicals and microbial pollutants. Despite growing awareness, there still are many unanswered questions, especially about the impact of long-term exposure and how atmospheric conditions affect the spread of MPs/NPs. The aim of this review was to bring attention to the issue of airborne MP/NP effects and to promote the development of advanced monitoring systems, a new multidisciplinary scientific field for the study of these novel pollutants, and global regulatory frameworks.

## 1. Introduction

Microplastics (MPs) are plastic particles smaller than 5 mm, exhibiting a variety of shapes that are produced by the use of plastic products, additives, cosmetics, etc. Nanoplastics (NPs) are tiny plastic particles with a size in the range of 1–100 nm [1,2]. According to a report by the United Nations Environment Program (UNEP), by 2050, there will be almost 13 billion tons of plastic litter produced, which will end up in landfills and natural areas [3]. Nevertheless, in 2023, following the COVID-19 pandemic, fossil fuels accounted for plastic production reaching over 413.8 million tons globally, according to the Plastics Europe study [4]. In that same year, fossil fuels accounted for 90.4% of the global production of plastics. The percentages of plastics produced worldwide that are post-consumer recycled and bio-based are 8.7% and 0.7%, respectively.

MPs in aquatic and marine environments are the subject of more than 96% of studies. Recently, airborne MPs have been identified as potentially harmful to the environment and human health. MPs can contribute to environmental contamination by being inhaled by humans or animals or by being deposited on surfaces. Research on airborne MPs is still in its infancy, mostly due to a lack of suitable technical tools for their detection and measurement [5,6,7,8]. Atmospheric transport mechanisms exacerbate the situation by dispersing MPs over long distances [9,10]. Wind currents, for instance, have the ability to carry MPs to far-off places with no direct human activity, such as pristine ecosystems like mountain peaks or polar regions. A cyclical pollution process that is linked to dangers to human health is further facilitated by secondary contamination routes created by airborne MP deposition on surfaces, such as soil and water bodies [11,12]. Although a great deal of effort has been put into comprehending the distribution, fate, and ecological effects of MPs in outdoor settings, comparatively less is known about their presence in the atmosphere, especially in interior environments [13]. Since the average person spends 90% of their time indoors, indoor spaces—such as homes, workplaces, schools, labs, and recreational areas—are the main hubs for human activity. Numerous plastic materials and consumer goods (i.e., furniture, carpets, textiles, electronics, and personal care items) found in these indoor areas have the potential to release MPs [14,15,16]. Airborne MPs must be sampled using extremely complex methods that need specialized equipment and expertise in addition to being very costly [6]. While sample collection and analysis are labor-intensive and time-consuming, identification and quantification usually require the use of spectroscopy and microscopy techniques, which come with high equipment, maintenance, and consumables costs [17]. Conventional methods for sampling airborne MPs usually include fixed-site monitoring, which might not offer geographic representativeness [18,19].

Airborne MPs provide unique health risks compared to those present in other environmental media. Airborne MPs can be inhaled and cause respiratory problems and systemic health impacts in humans, even though they mainly harm aquatic life and terrestrial creatures. Recent research has shown that breathing in MPs can trigger inflammatory reactions and may contain harmful chemicals and bacteria that could exacerbate pre-existing medical conditions [20,21]. The accumulation of MPs/NPs in human lung tissue has been demonstrated by studies in the literature, but the long-term effects of this exposure are yet unknown. Elevated MP concentrations in industrial settings may be linked to respiratory conditions like pneumonia and perhaps cancer. However, there is insufficient information to draw a clear link between environmental contaminants and plastic additives. These contaminated MPs can increase the risks to one’s health by releasing the absorbed harmful substances into the lungs or other regions of the body when inhaled [20,21].

**Figure 1:**
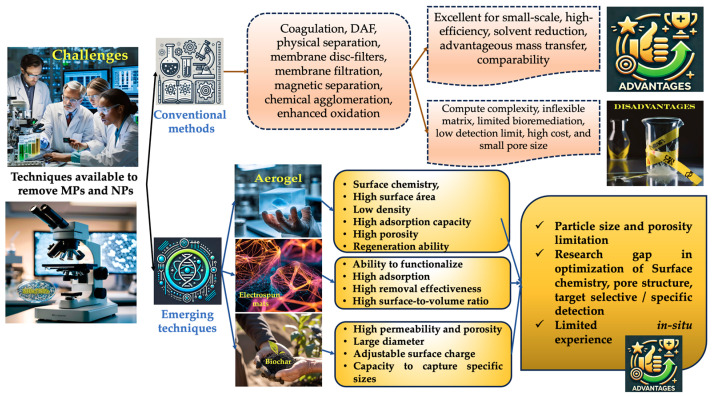
An overview of the methods used to study MPs/NPs.

Research on identifying and quantifying MP sources in ambient air is lacking. Airborne MP dispersion models could be one approach to solving this issue by using mathematical and numerical methods to replicate the primary physical and chemical mechanisms that dictate the movement and dispersion of pollutants in the atmosphere [22]. The properties of pollutants in the atmosphere and their air concentrations are characterized by advanced MP dispersion models using meteorological factors such as input data, along with topography data, land use, and source characteristics [20,23] (Figure 1). To the best of our knowledge, there are no comprehensive assessments of airborne MPs’ exposure in the current literature. The majority of studies on airborne MPs have just documented their properties and abundance, with little effort made to identify possible sources using a range of methods. The objective of our review was to provide a comprehensive analysis of all factors associated with MP pollution in ambient air, both indoors and outdoors. In order to identify important information gaps and suggest recommendations for future research and policymaking in this area, a major part of our review was to compile and contrast the methods and techniques currently used for MP/NP sampling and identification (indoors and outdoors MPs/NPs) [2,24]. In order to close the evident knowledge gap on MP/NP contamination in indoor and outdoor settings, we also want to provide an overview and critical analysis of the issue of airborne MPs/NPs [25].

**Figure 2:**
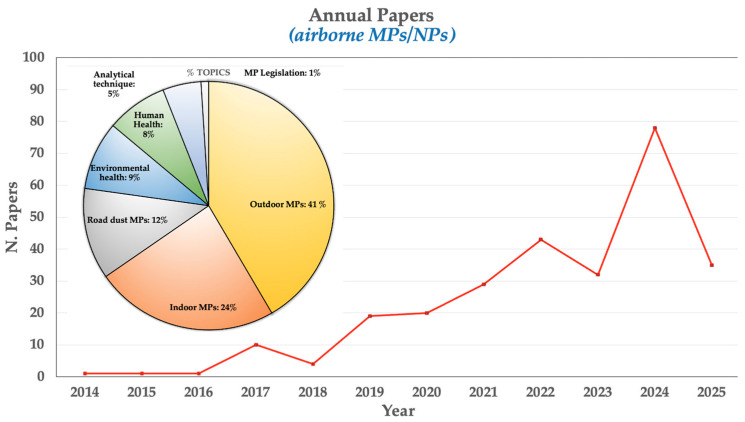
An illustration of the temporal analysis of articles in airborne MPs/NPs research.

This study was based on research published in PubMed, ISI Web of Science, Google Scholar, and Scopus until March 2025. The following pair-wised keyword combinations were adopted to search the documents: airborne microplastics AND airborne nanoplastics AND indoor MPs/NPs OR outdoor MPs/NPs OR neurotoxicity OR oxidative stress OR biocorona OR cellular uptake OR carcinogenesis OR neurodegeneration OR inflammation. The complete texts of the relevant articles were examined and evaluated by each contributor. The reference lists of the publications that were obtained were also examined and discussed in order to find the most pertinent research. Only English-language papers published in peer-reviewed publications were taken into consideration for review. Figure 2 shows a summary of every study (*n* = 272) in the literature that was considered for the review.

By providing a comprehensive and multidisciplinary examination of airborne MPs/NPs, this review marks a significant advancement in the scientific literature. It is notable for its depth, comparative approach, and future recommendations. Technical details, advantages, operational limitations, equipment requirements, and suitability for both indoor and outdoor settings are discussed. Unlike previous research that solely examines one strategy, this review provides a crucial comparison reference for selecting methods based on the study situation. Additionally, this review provides one of the most comprehensive analyses of potential health impacts of MP/NP inhalation to date. Oxidative disruption, lysosomal damage, biocorona formation, and neurotransmission interference are some of the modes of action. This integrative approach (Multidisciplinary Integration of Toxicological Risks) is consolidating the new field of Medical Polymer Science, which integrates materials science, medicine, cell biology, and environmental toxicology.

## 2. Sources of Airborne MPs/NPs

MPs/NPs are widely distributed, as evidenced by the finding of their pollution in fish, soil, sediments, rivers, lakes, and snow. The fate of microplastics is greatly influenced by the atmosphere, which serves as a crucial transporter [26]. Following a thorough analysis of all the data presented in the literature, all of the research on airborne MPs—both indoor and outdoor—is shown in Tables S1 and S2. Through bubble ejection, wave action, or wind, MPs/NPs that have accumulated in the environment can become sources of MPs/NPs in the atmosphere. The MPs/NPs introduced into the marine atmosphere by marine and land-based emissions turn the ocean from a sink into a source of atmospheric MPs/NPs. Previous studies have shown that the wind erosion can release MPs and NPs that have accumulated in soil into the atmosphere [5,27]. Up to 48,000 tons of MPs/NPs are thought to be released annually from soils worldwide through dust. Investigations have also been conducted on the theoretical underpinning for MP enrichment rates at the soil–air interface. However, not enough knowledge about the transfer mechanism limits the ability to predict emissions [26,28,29,30].

In contrast to the limited primary emissions from human activities in remote regions, the resuspension of accumulated MPs/NPs in soil might be a significant source of MPs/NPs in the atmosphere. MP contamination in road soil is also a result of road markings and channeling tools (cones, drums, and barrels). Plastic paints are commonly used to paint road markings, especially pedestrian crossings. According to some research [31,32], road markings are usually composed of two separate layers: the base layer gives them color and makes them visible during the day, and the glass microsphere layer allows for retro-reflection and makes them visible at night under car headlights. As previously mentioned, a number of factors, including weathering, physical oxidation, chemical oxidation, rise of temperature, and exposure to UV radiation can cause MPs to disintegrate and release into the atmosphere. These factors promote oxidation of the polymer matrix, which in turn causes the breakdown of covalent compounds [33,34,35]. The physical and chemical characteristics of plastics, including color, surface morphology, crystalline quality, particle size, density, reactivity, surface functionality, and hydrophobicity, are impacted by the degradation processes. UV radiation makes it easier for polymers to undergo chemical changes. For instance, ruptured enlaces of polyaldiglycol carbonate and hydroxyl groups were formed when exposed to UV radiation with wavelengths of 253.7, 300, and 350 nm [36].

These activities occur more quickly in the atmosphere than in the marine environment because of the higher oxygen content and stronger UV light. Ozone depletion accelerates the deterioration of dust particles, but glaciers’ low temperatures, which allow the ozone to persist over a large area, slow down the rate of degradation [37]. It is believed that small particles are less susceptible to physical impact, and that weathering mechanisms are dependent on particle size. UV radiation and air oxidation are thought to be responsible for the majority of weather damage incidents [38,39,40]. The age of dust particles has been estimated using microscopic methods that identify bubbles, cracks, breaks, and bifurcations of fibers caused by mechanical erosion (for example, by collisions with sand grains in the air). Discoloration due to chemical oxidation may indicate the prolonged exposure of dust particles to the air, while the smooth edges and bright color suggest recent formation [41].

### 2.1. The Common Type of MPs/NPs (Fibers, Fragments, Microbeads)

The primary source of MPs in the atmosphere is synthetic textiles, the production of which has been rising significantly on a global scale [21]. Various fibers made of polymers, such as polyester (PES), polyamide (PA/nylon), polypropylene (PP), and polyacrylonitrile (PAN), are common polymeric materials used in synthetic textiles. A significant quantity of fibers is shed as a result of cutting and abrasion in the textile industry as well as everyday garment wear, washing, and drying; thousands of fibers are released from a single gram of fabric [21,42,43]. Due to their wide availability and ease of dispersion, synthetic textile emissions account for a significant quantity of MPs in urban settings [44,45].

MPs in the atmosphere can reach up to 322 km from the surface of the earth while still hanging, according to recent studies. It should be noted that this dispersion range may change based on source-specific, hydrological, and geographic factors [46]. According to their shape, MPs can be categorized into multiple groups and display a variety of morphologies [1]: fibers are thread-like particles with a minimum length-to-width ratio of 3:1 and uniform thickness throughout; fragments are irregularly shaped particles with a maximum length-to-width ratio of one and a minimum height-to-width ratio equal to the width-to-length ratio; microbeads are spherical plastic particles, such as granules; films are thin, flat particles with a significant surface area. When MPs break down into their original plastic product forms, such as fibers for synthetic fabrics and foams for PS packaging, it is helpful to look for a possible source of these particles based on their morphology [47,48]. Synthetic textile items include apparel, home textiles, awnings, sails, ropes, carpets, and medical masks as the source of microfibers [49,50]. Certain fibers, like glass, rubber, and cigarettes (a cigar filter contains around 15 million acetate fibers), are not textile-based. In metropolitan environments, fibers are the most common morphotype, both indoors and outdoors, as well as in isolated locations [51,52,53,54]. The primary cause of films is the breaking apart of plastic bags and other packaging materials. The foamed polystyrene (PS) films are made from PS products that are primarily used for containers and packaging materials. They are widely employed in the rapid delivery business for both packaging and thermal insulation [45,55,56].

Microscopic techniques that detect bubbles, cracks, splits, and bifurcations of fibers brought on by mechanical erosion—for instance, by collisions with sand grains in the air—are used to determine the age of dust particles. While the bright hue and smooth edges suggest recent production, discoloration from chemical oxidation may indicate dust particles have been exposed to the air for a long time [35,46]. Polymer composition, in addition to particle form, aids in determining the source of airborne MPs. Fleeces, tents, rain gear, and climbing ropes are all made of polypropylene (PP) and polytetrafluoroethylene (PTFE). One common material for packaging is polyethylene (PE). Additionally, PP is used to create fibers for the textile industry, disposable plastic cups, and bags for food packing and medical supplies. Polyethylene terephthalate (PET) possesses consistent mechanical and physical characteristics, is resistant to disintegration, and is robust even at 120 °C [55,57,58].

One of the most widely used plastics for making textiles and plastic bottles and packaging is PET. Surprisingly, bottle manufacturing only constitutes 30% of the global demand for PET, whereas the majority of PET production (more than 60%) is utilized for synthetic fibers [59]. PAN is commonly used to strengthen the mechanical qualities of cement mortar materials and is utilized to create knitwear and outdoor textiles (awnings, sails, etc.) because it provides exceptional UV protection. PA is used in textiles, medications, beverages, furniture, household appliances, transportation, candles, toothbrushes, packaging, carpets, automobile parts, and fishing gear since it is four to five times stronger than wool and ten times more resistant to wear than cotton [60]. Low-density polyethylene (LDPE) is frequently used to make packaging and trash bags because of its flexibility, low cost, outstanding chemical resistance, and lightweight characteristics. High-density polyurethane (PU), which is used to make building foams, quickly loses its qualities when exposed to UV light. The construction sector, car tires, and textile fibers are the most probable sources of polyvinyl chloride (PVC) [55,59,61].

### 2.2. Road Dust

The transportation industry is a source of airborne MPs/NPs. A significant contributor to air pollution is non-exhaust emissions (NEE) of particulate matter from brakes, tires, road surfaces, and road markings [43,62]. Heterogeneous road dust is created when particles that have been deposited on the road surface mix and are resuspended by traffic. From 2.11 × 10^6^ tons in 2012 to 2.89 × 10^6^ tons in 2019, cars have become a larger global contributor to tire wear MP generation. An additional 0.5 million tons of MPs are contributed by brake wear particles [32]. Furthermore, particles from non-traffic sources, like pollen, spores, organic matter released during biogenic activity, mineral dust from adjacent soils, construction sites, quarries, and pollen, frequently land on the road surface and can interact with traffic-derived NEE.

One factor contributing to MP emissions is vehicle wear, which includes friction from tires, brakes, road surfaces, and airplane tires. According to current theoretical estimations, tire wear particles (TWPs) are a significant source of MP pollution worldwide and are frequently regarded as microplastics [43]. TWPs can settle at the roadside or enter the environment by runoff or air when they are created on the road surface by the tire–pavement interaction [62]. According to a number of studies, the majority of coarser particles end up in the neighboring ditch or by the side of the road. Finer particle fractions can then be carried by air movement or runoff into storm water systems. Nevertheless, there is still a dearth of empirical data and a knowledge gap regarding the presence and movement of TWPs from the road surface into the environment [62]. Moreover, additional elements, such as particulate matter (PM), or more specifically, minerals or metals, have seldom ever been included in the same tests when looking into PMs and TWPs. However, to obtain a thorough understanding of the contribution and transport of traffic-derived pollutants, it is crucial to analyze the wide range of traffic-derived particle types (such as PM, metals, and organic pollutants) in ambient samples from road environments. This is because traffic-derived PMs mix with particles from more remote sources as well as with each other. MPs in roadside ditch sediments and water have mean sizes of 60 and 100 µm, respectively.

TWPs can be created by abrasion and are categorized as coarse (PM10) particles, which range in size from 10 to 2.5 µm; fine (PM2.5) particles, which range in size from 2.5 to 0.1 µm; and ultrafine particles (PM0.1), which are smaller than 0.1 µm. The majority of TWPs fall into the coarse fraction [32,43]. Previous research has shown that about 52% of the natural or synthetic polymers found in tire wear and road abrasion include different plastic components including styrene-butadiene rubber. This implies that a sizable amount of plastic particles, together with specific concentrations of antioxidants, desiccants, plasticizers, and other additives are released into the environment annually throughout this release process [43].

Previous studies in the literature have shown that road traffic alone can release about 12,301 MP particles each day. Every year, millions of tons of MPs are produced in Europe as a result of tire and road wear particles. According to [59], these particles can damage the air by direct discharge or resuspension in road dust; they also account for about 11% of PM emissions worldwide. Recent studies’ estimates indicate that tire and road wear particles account for almost half of PM emissions in Denmark and Norway and roughly 30% in Germany [63]. Road wear particles are composed of a range of wear particles, including those from tire wear, road wear, and brake abrasion [63].

### 2.3. Outdoor/Indoor MPs/NPs

**Figure 3:**
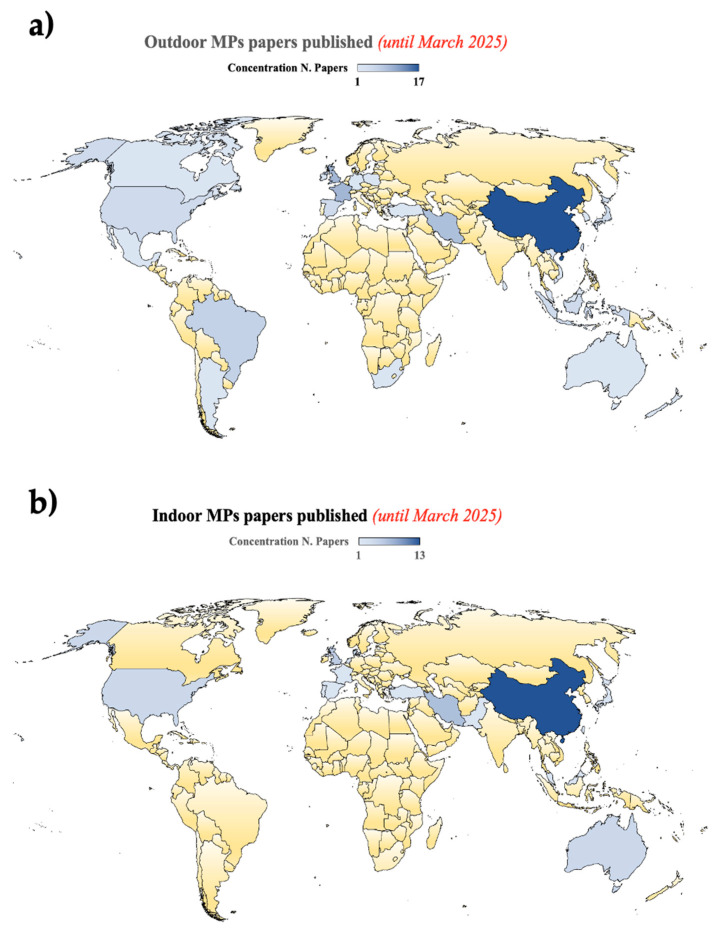
Countries that have published about outdoor MPs (**a**) and indoor MPs (**b**).

As was previously discussed in Section 2 of this review, MPs/NPs in outdoor contexts emerge from a number of sources, including the fragmentation of large plastics (bags, containers, etc.), which decompose when exposed to sunlight, wind, and rain [64,65,66,67]. The research on outdoor MPs/indoor MPs published up until March 2025 is displayed in Figure 3. Additional factors include tire wear from driving on asphalt, which releases MPs/NPs into the air and drainage systems; industrial processes that release MPs/NPs into rivers and soils, contaminating large areas of land and water; airborne particles that can travel long distances and settle in soils and water bodies, etc. [68,69]. Outdoor MPs/NPs can take many different forms, including resin pellets used as raw materials for the production of plastics that are frequently spilled into the environment, microfibers from the breakdown of synthetic textiles, microspheres used in industrial cleaning products and some cosmetics, and fragments from broken and degraded plastic objects. PE, PP, PS, PU, PVC, and styrene butadiene (SBR, as a result of tire abrasion) are the primary outdoor MPs [66,70,71,72].

Figure 3a,b highlight the main countries with the greatest number of studies on outdoor and indoor MPs/NPs, respectively. The nations mentioned in the research studies are where the MP samples were gathered. According to our bibliometric analysis, China leads the world in the number of publications on airborne MPs, because of substantial national funding, whereas Iran ranks highest among Asian nations (aside from China), indicating a growing interest in the region. The US and the United Kingdom, which are notable for their substantial output in this field, follow next. Australia, Spain, India, and Germany have also made substantial contributions to the global research body. The bulk of research on outdoor habitats has focused on urban, industrial, and agricultural environments, where human activity has resulted in greater MP concentrations, based on a comparison of the studies that have been presented. MP concentrations in indoor environments—which have received less attention—can be comparable to or even higher than those outdoors, especially in enclosed and poorly ventilated places, because of sources such as synthetic textiles and furniture. In conclusion, over the past 15 years, research on airborne MPs has increased significantly, with China, the US, and the UK leading in this field.

Household dust containing MPs/NPs from furniture, carpets, and cleaning products; personal care products (i.e., exfoliants, creams, and makeup) that may contain MPs/NPs in the form of microbeads; and synthetic clothing (i.e., washing and wearing clothes) that releases plastic microfibers into the air and water are some of the sources of MPs/NPs that litter indoor spaces [73]. Additional sources include furniture and decor (such as sofas, curtains, and other synthetic polymer goods), plastic containers and kitchen utensils (i.e., heating and wear of plastics in the kitchen), and other objects that gradually release MPs and NPs [72,74,75]. Dust, which is essentially a mixture of synthetic fibers, pieces, etc., and microfibers, such as those found in clothes and upholstery, are the most prevalent indoor micro-nanoparticles. MPs/NPs can be inhaled or consumed through food and water in indoor settings, which is another factor to take into account. According to various studies in the literature, extended exposure may be associated with respiratory issues as well as other negative health impacts, such asthma, COPD, fibrosis, etc. [76,77,78,79].

**Figure 4:**
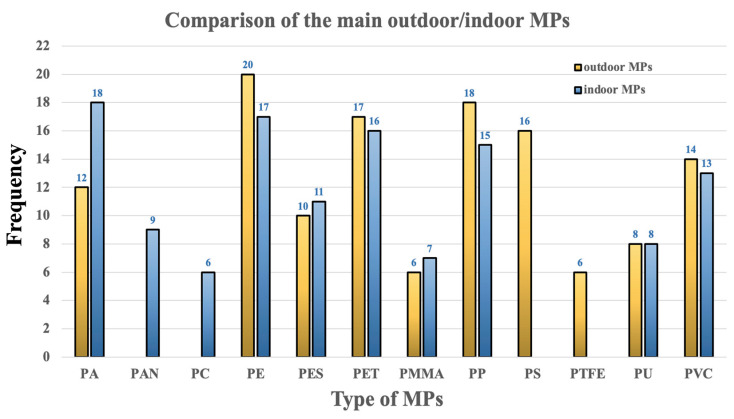
Comparison of the main outdoor/indoor MPs.

As previously indicated, the indoor environment is dominated by plastics from household products, items for kids, and synthetic textile fibers. PES, PET, PA, PAN, PU, PP, PE, and PVC are the most widely used MPs/NPs. PVC is a common plastic used in construction and household goods. It can be found in toys (i.e., wear), cables and electrical insulation (i.e., degradation of plastic covers over time), ventilation and air conditioning systems (i.e., accumulation and redistribution of MPs in the air), as well as others [21,80,81,82]. It has been found that the chemical makeup of MPs/NPs detected in both indoor and outdoor settings might vary depending on the source of contamination and the surrounding circumstances. Although MPs/NPs’ contamination affects both indoor and outdoor environments, indoor exposure may be more severe because of the increased concentrations and buildup of microfibers in home dust and the air [83,84,85,86]. Figure 4 illustrates a comparison of differences and similarities in the makeup of airborne outdoor/indoor MPs.

A comparative graph shows the estimated frequencies of the primary airborne outdoor/indoor MP types identified in the studies examined in this review. In both scenarios, PA, PE, PET, and PP predominate, while their frequency varies. Millions of commonplace items have these four ubiquitous polymers. Both use (indoors, such as in bags, wrappers, synthetic clothing, carpets, and food containers) and exposure (outdoors, such as in packaging, plastic debris, medical supplies, and textiles) release them into the environment. They fragment instead of decomposing readily. These polymers are not particularly dangerous by themselves; what is most concerning about them is the combination of their size, the pollutants they contain, and the additives they include. Long-term exposure can change hormone balance, raise the risk of inflammatory disorders, and cause oxidative stress in vital organs including the liver, lungs, or intestines (bioaccumulation). For instance, PA is most prevalent in the interior, which is consistent with its extensive use in carpets and textiles. PA microfibers do not readily evaporate and either stay in the air or settle on surfaces as dust. PA fibers have a lower lifespan in outdoor settings because they deteriorate more quickly in the presence of wind, rain, and UV radiation. Depending on the route of intake, PA may have an impact on human health; inhalation may result in allergies, asthma, inflammation, and irritation. Consumption may encourage potential immunological, gastrointestinal, and hormonal changes. People with asthma and chronic obstructive pulmonary disease (COPD), those who work in the textile or recycling industries and are frequently exposed to synthetic fibers, and children are typically the most vulnerable, because they breathe more air relative to their weight and because they crawl on the ground.

In contrast to the four main MPs previously discussed, some MPs are found in a particular environment. PC and PAN, for example, are found indoors but not outdoors. PAN-MP, a polymer found in homes, workplaces, and enclosed structures, is frequently utilized in synthetic textiles (such as carpets, blankets, and clothes). PC-MPs are commonly utilized in office supplies, electronics, eyeglasses, and other products. Regular use and deterioration result in the production of microfibers and microparticles. PAN-MPs are probable carcinogens that can irritate the respiratory system and perhaps induce neurotoxicity. Bisphenol A (BPA), an endocrine disruptor that may be linked to problems with metabolism, development, and reproduction, is released by PC-MPs.

The majority of PS-MPs and PTFE-MPs are found outdoors. PTFE is utilized in industrial coatings, pipes, seals, and non-stick coatings because of its exceptional heat resistance. PS is frequently used in packaging, including as food trays and throwaway cups. These products are disposed of outside and are vulnerable to weathering and UV radiation deterioration. PS fractures readily, producing MPs in parks, streets, and landfills. High-temperature hazardous gases, including perfluoroalkyl and polyfluoroalkyl compounds (PFAS), are produced during the breakdown of PTFE. Perfluorooctanoic acid (PFOA) is an example in this class of chemicals. As PS breaks down, styrene—a potentially carcinogenic substance—may be released, causing liver damage, lung discomfort, and neurological disorders.

## 3. Key Sampling Techniques for MPs/NPs as Well as Challenges with Identification and Quantification

Below is a detailed discussion of the main sample methods for MPs/NPs, as well as the challenges associated with their detection and measurement. As mentioned in Tables S1 and S2, the present review attempts to highlight the salient aspects and limitations of each technique used in the chosen studies.

### 3.1. Sampling Techniques

Both active (electric pumps) and passive approaches are used to collect aerosols from the air both indoors and outdoors in order to sample MPs and NPs [87,88,89]. The methodologies used depend on the type of matrix being analyzed, the MPs’ size, and the equipment available [90,91]. Passive samples ranging from 1.0 × 10^−4^ to 3.3 × 10^−1^ m^2^ have been used with samples of dry and wet atmospheric depositions [92,93,94,95]. A vacuum cleaner, hog bristle brush, or outdoor broom with a wooden brush and metallic pan are used to gather indoor (floor) dust samples. The active sampling technique is utilized to detect the presence of suspended MPs/NPs. Active sampling pumps use a variety of filter paper types, such as glass microfiber filters (pore size 1.6 mm), PTFE (pore size 2 mm), and quartz filter paper (pore size 1.6 mm). The frequency range for the sample collection is 4–40 h, and the air volume measured is 2–24 m^3^ [96,97,98].

### 3.2. Separation Treatments

A sample treatment is typically used before the analysis in order to achieve MP/NP purification, which eliminates unwanted material. Two popular methods for purifying MPs/NPs are density separation and enzymatic digestion. There are two kinds of digestions: acidic (H_2_O_2_, HClO_4_, HNO_3_) and basic (Fenton’s reagent, or enzymatic) [99]. After digestion, density separation by ZnCl_2_, NaI, and NaCl treatments may be used to further separate the MPs/NPs from the other particles [100]. Since NaI has some of the greatest efficiencies for the collection of denser polymers, it is most often used [101]. Nevertheless, in the case of samples of the airborne MPs/NPs, the filters are much cleaner than the sediments and dust samples. So, no or less preprocessing is needed for airborne MP/NP samples.

### 3.3. Visual Identification

Microscopic techniques are used for a first characterization of the MP/NP shape, size, and color. Stereomicroscope is the preferred method since it is relatively simple, fast, and inexpensive. However, it also has limitations like its lower resolution and difficulties in examining colorless, transparent, and spherical particles [102]. Nonetheless, optical and electron microscopes can be used for better resolution. Synthetic MPs/NPs cannot be identified by standard microscopes. The usage of Nile red as a fluorescent dye for staining MPs/NPs is effective for the identification of various polymers, but it may result in false positives [103,104]. Though more expensive, scanning electron microscopy (SEM) is used to characterize the microscopic structural features of the MPs/NPs of the particle surface [105]. Besides the extensive preparation, a drawback is that the electron beam used in SEM can potentially damage delicate samples, especially MPs/NPs with smaller sizes [106]. It should be noted that SEM is unable to provide information on the particle composition. After the morphological analysis, the MP/NP analysis is carried out on quantitative and qualitative aspects by thermochemical and spectrometry methods [107].

### 3.4. Thermochemical Analytical Methods

Because of their accuracy and efficiency, pyrolysis-gas chromatography mass spectrometry (Py-GC/MS), thermal extraction desorption-gas chromatography mass spectrometry (TED-GC/MS), and matrix-assisted laser desorption ionization time of flight mass spectrometry (MALDI-TOF-MS) analytical techniques have significantly improved the detection and characterization of MPs/NPs [108]. These methods can infer the composition and structure of MPs/NPs and give the mass fraction. The most frequently used method is Py-GC/MS, which entails breaking down organic molecules thermally without oxygen, then using gas chromatography mass spectrometry to separate and identify the resultant chemicals. Due to its high sensitivity, Py-GC/MS can analyze trace amounts in airborne MPs/NPs with a limit of detection lower than 1 µg [109,110]. However, Py-GC/MS cannot identify plastic particles smaller than 50 µm (longest dimension) due to the necessity of manually placing the sample into the heated tube [111]. Moreover, the co-pyrolysis interaction could affect the accuracy of quantitative results [112,113,114]. An alternative method to address these issues is the thermogravimetric-spectrometry, which tracks and analyzes both the pyrolysis process and the products [115,116]. TED-GC/MS involves the thermal extraction and desorption of volatile and semi-volatile molecules, which are then analyzed by GC-MS. It does not require a lot of sample preparation and is non-destructive. It offers a reliable method for identifying the MPs/NPs of varying polymer compositions [117]. MALDI-TOF-MS uses a laser to ionize the sample in a matrix, and the ions are separated based on their mass-to-charge ratio in a time-of-flight mass analyzer to detect very small MPs and NPs. It is excellent at identifying and distinguishing polymers. MALDI-TOF-MS enables quick analysis with minimal sample preparation [118]. Recently, a fusion and solvent evaporation ionization (FSEI) device, comprising a heated plate and nebulizer, for the direct detection and identification of MPs/NPs by mass spectra has been developed (FSEI-MS) [119].

### 3.5. Spectroscopy Analytical Techniques

Nowadays, MP/NP studies frequently use Fourier-transform infrared (FTIR) and Raman spectroscopy. FTIR utilizes infrared absorption spectra to identify MPs/NPs. FTIR spectroscopy is often employed in conjunction with microscopy, and offers comprehensive details regarding the distribution, morphology, and chemical makeup of MPs/NPs. Because MPs/NPs have a distinct molecular structure， FTIR spectroscopy can produce a unique spectrum that can be used to determine the functional groups of molecules and polymeric makeup [120]. Even with its great sensitivity, specificity, and non-destructive properties, FTIR is still a laborious analytic method that requires extensive preparation. Pollutants may affect FTIR spectra, making it more appropriate for indoor samples [121,122]. Attenuated total reflection (ATR), reflection, and transmission are the three primary FTIR modes that have been identified. Since ATR-FTIR requires crystals like diamonds to be in touch with the MP surface, it is usually utilized to analyze larger MPs (>500 µm) [123].

The detection accuracy is much worse when using samples <50 µm, fibers, fragments, and thin films as examples of small plastic particles that can be analyzed using the transmission mode. This makes it possible to distinguish between different kinds of polymer using their distinctive infrared absorption spectra [124]. When examining opaque MPs/NPs that are challenging to arrange, like thin, uniform layers, such as particles or films on surfaces, the reflection mode works well. Rapid sample screening is another use for this technique [125]. There have been reports for two common resolutions: 4 and 8 cm^−1^, which normally call for 16 and 64 scans. Because LOD of the FTIR (10–20 µm) is typically larger than Raman spectroscopy, the latter is generally preferred for analyzing smaller particles. Micro-Raman spectroscopy can examine particle size, shape, and morphology, as well as important details about the chemical composition, surface chemistry, and functional groups [19,23,126]. In contrast to FTIR, Raman spectroscopy measures the scattering of light by the sample, to identify changes in the polarizability of molecules during vibration [91,127]. The smallest limit size for Raman is 1 µm [128]. By using laser stimulation near to the absorption bands of the sample, resonance Raman spectroscopy dramatically increases Raman signals while reducing background fluorescence. There are two main laser wavelengths (532 nm and 785 nm).

A number of investigations use two laser wavelengths, but the majority only utilize one [94,129]. Achieving high-quality analytical results requires increasing the signal-to-noise ratio by adjusting the laser acquisition (ranging from 2 to 10 s) and accumulation (up to four) to improve the spectrum for particles <500 nm [130]. Raman is non-destructive and time-consuming, just like FTIR. On the other hand, Raman offers excellent high spectral resolution, with an accuracy of less than 1 µm [128]. Furthermore, intense Raman scattering from organic compounds and additives may overlap with the polymer’s characteristic peaks. Additionally, the capture of unambiguous spectra from MPs is complicated by the susceptibility of Raman signals to sample fluorescence interference [131]. A Raman analysis is appropriate for examining indoor samples due to its properties. While conventional FTIR and Raman spectroscopy can identify microplastics in total suspended particles, advanced instruments such as µFTIR, atomic force microscopy-based infrared (AFM-IR) [132] are useful.

Micro-Raman microscopy (µRaman), and SEM are crucial for analyzing inhalable MPs/NPs (e.g., particles smaller than 10 µm) [133]. µ-FTIR provides information about a specific point of the sample, so it can automatically identify MPs as small as 10 µm and NPs on filter membranes without sorting. Atomic force microscopy-based infrared spectroscopy (AFM-IR) is a promising technique that allows for the analysis of NPs [41,134]. µRaman is a combination of a conventional light microscope with Raman spectroscopy that makes it possible to analyze NPs in a very efficient “point and shoot” manner [135,136].

SEM is a microscope technique that uses a focused beam of electrons to obtain high-resolution images of the surface of a sample. The minimum detectable size for SEM scanning is 1 nm [137]. EDX is an analytical technique used in conjunction with SEM (i.e., scanning electron microscopy with energy dispersive, X-ray analysis, SEM-EDX) to identify the elemental composition of materials based on the characteristic X-rays emitted when a sample is bombarded with electrons. The SEM-EDX analysis provides a fast, non-destructive elemental composition of particles using an electron beam. Based on the spectra, this method allows for the detection of particle contamination, along with the determination of the degree of weathering and oxidation of MPs/NPs [42,138].

Selecting and interpreting any analytical technique for airborne MPs/NPs require a thorough understanding of the interactions between the matrix (i.e., environmental compartment), sample size, and analytical equipment. Dry and wet atmospheric depositions (passive sampling), indoor dust (passive “mechanical” sampling), and suspended particulate matter (active air sampling) are the three primary matrices and the corresponding sample size ranges. The complexity of sample preparation and representativeness (deposit vs. dust vs. air) are determined by the kind of matrix, whereas the analytical technique and detection limit are determined by the sample size (area vs. volume vs. mass). For atmospheric MPs/NPs studies, it is crucial to understand these interactions in order to develop reliable and comparable sampling techniques. Given the limitations of each approach for selecting the best sampling strategy, it can be claimed that atmospheric deposition is location-dependent and affected by local conditions (such as wind), which may skew spatial representativeness and make it impossible to identify daily or hourly variations. It can be applied to remote areas where active sampling would necessitate a significant logistical effort as well as long-term flux studies (i.e., measuring the annual MP deposition). The exceedingly heterogeneous matrix (a mixture of organic components, minerals, and MPs/NPs) is a limitation of the indoor dust technique (passive mechanism); it requires density separation and chemical/enzymatic digestion, which lengthens the analysis time. In toxicological studies that require huge sample loads for biological tests, it is helpful for estimating domestic human exposure, since it represents the real particle reservoir to which we are exposed. High-flow pumps and a power source are necessary for the examination of suspended particles (active filtration sampling), which can be less practical in remote areas. Samples with low MP/NP concentrations may not have enough mass for a thermochemical analysis (i.e., Py-GC/MS). Studies with temporal resolution (such as traffic peaks or weather events) and direct comparison with PM_2.5_/PM_10_ standards, as well as the assessment of the inhalable proportion, benefit from this technique. Despite its lack of temporal accuracy, the deposition plate sampling approach is simple and inexpensive, making it suitable for deposition flux analysis and worldwide studies. Since household dust sampling reflects the reality of MPs/NPs being inhaled and consumed, it may be suggested as a means of assessing indoor human exposure, notwithstanding the cost of pretreatment. Active sampling with standardized filters can be utilized to quantify MP/NP concentrations in air and their fluctuations, particularly if time series and MPs/m^3^ data are required. For temporal resolution, spatial representativeness, and public health significance, it is frequently ideal to use a combination of techniques (i.e., active sampling for monitoring and passive sampling for long-term flows).

### 3.6. Novel MPs/NPs Detection Methods

FTIR and Raman spectroscopy offer molecular specificity but struggle with lower particle sizes. Thermal analytical methods like Py-GC/MS provide compositional insights but are damaging and limited in the morphological analysis [135,139,140]. To help with these issues, new approaches have been developed in the last two years, using concepts like voltammetry and impedance, and electrochemical, new (bio)sensing technologies by providing sensitive platforms for detecting MPs/NPs. Through nanostructure-enhanced detection, plasmonic techniques such as surface plasmon resonance (SPR) and surface-enhanced Raman spectroscopy (SERS) offer great sensitivity and specificity [141]. Another promising approach is using carbon dots, which can interact with gas molecules, and they can be used to create efficient gas sensors that track airborne MPs/NPs [142,143,144,145].

Airborne MPs/NPs can be found via autofluorescence, which examines how they glow in specific conditions and by laser-induced breakdown spectroscopy (LIBS) [146,147,148]. Laser-induced breakdown spectroscopy (LIBS) is an atomic emission spectroscopy technique that uses a laser to excite a sample to produce plasma, and the composition and content of elements are determined by analyzing the resulting spectrum. The rapid detection and multi-element analysis of LIBS show its unique advantages in the detection of atmospheric particulate matter [146,147,148].

## 4. Environmental Risk of Airborne MPs/NPs

Airborne MPs/NPs are micro-nanoscopic particles that are becoming an increasing environmental issue due to their dimension and ability to migrate long distances on wind. Plant life and soil organisms may be affected by airborne MPs and NPs that land on terrestrial surfaces and build up in the soil. Weathering may cause them to further degrade over time, but their toughness and small size allow them to endure for extended periods of time. In a similar vein, these neo-pollutants can reach water bodies including rivers, lakes, and oceans by wind or rain [149,150,151]. They can damage aquatic life and make their way into the food chain once they are in an aquatic environment. Wide-ranging effects on marine ecology may result from a rising load of edible plastics in the air. A vast variety of aquatic creatures, including zooplankton, small fish, and larger species like sea turtles, seabirds, and whales, can consume MPs once they are in the water. Blockages in the digestive tract, a false sense of fullness that results in malnutrition, and toxicity from absorbed chemicals are all consequences of consuming plastics, which can contain pollutants like heavy metals and polychlorinated biphenyls (PCBs). Biomagnification is the process by which toxic compounds build up in organisms and ascend the food chain to eventually reach humans [72,152]. Prey-sized MPs and NPs can harm marine species, and consuming them may have unknown biological repercussions. It is interesting to note that MPs/NPs in the atmosphere and those consumed by different marine species have a similar approximate size range, suggesting higher bioavailability. Once in the hydrosphere, indigestible MPs/NPs from air deposition would have detrimental ecological effects on marine life. Future ecological concerns will be exacerbated by the growing oceanic burden of airborne MPs/NPs [9,153]. A rational risk assessment is facilitated by knowledge about origins of the MPs/NPs, distribution, and mode of transportation.

Although air transport is acknowledged as a significant route for terrestrial MPs to reach the ocean, little is understood about their global atmospheric contribution [9,153,154]. Crucially, the physical and chemical aging processes that affect the transport dynamics of MPs and NPs are not taken into account by existing modeling techniques [155,156,157]. A number of variables, including humidity, UV rays, secondary aerosol production, and bacterial activity, affect these aging processes. Our capacity to appropriately incorporate MPs/NPs’ aging processes into models is limited by the dearth of pertinent mechanistic data [158]. Consequently, the behavior and fate of MPs/NPs in the atmosphere may not be adequately captured by the results of existing models [9,159]. Atmospheric measurements cannot be globally harmonized or provide a worldwide flux of MPs/NPs’ surface emissions due to limited sensor technology [160]. Moreover, evaluating the emission flow using inverse modeling with little observational data is fraught with uncertainty. Remote areas are especially susceptible to the effects of small particles since smaller MPs/NPs are easier to carry over long distances and are therefore more equally distributed in the atmosphere. Large MPs/NPs particles, on the other hand, are more likely to be deposited near their emission source. Overall, a quantitative understanding of the behavior of MPs/NP particles in the atmosphere is necessary to mitigate the dangers that these emissions bring to ecosystems and human health [161,162,163,164].

**Figure 5:**
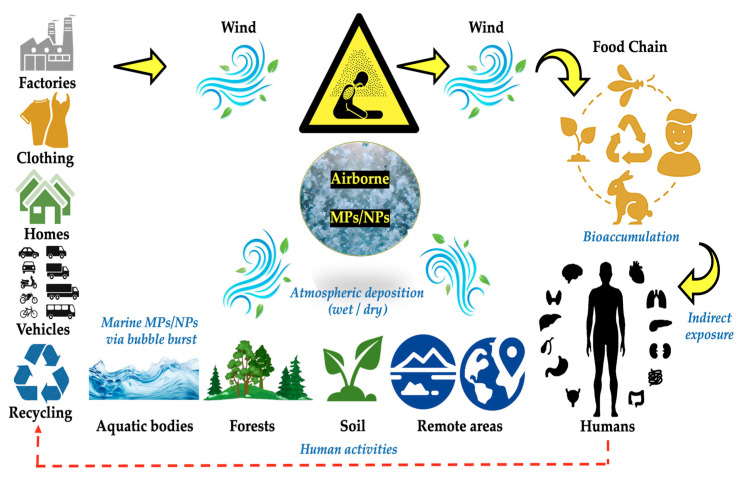
Potential origins and environmental distribution of airborne MPs/NPs.

Airborne MPs/NPs may be a contributing factor to atmospheric changes, according to certain research [164,165,166,167]. They might interact with pollutants or have an impact on cloud formation, which could change weather patterns. Nonetheless, this field of study is still in its infancy [72]. The MPs and NPs that are in the air can be hazardous and can potentially leak harmful substances. Plastic products, for instance, may include environmental chemical pollutants like pesticides or heavy metals that can be passed on to species that consume them. This has sparked worries about biomagnification, which is the buildup of poisons in the food chain, and which can endanger ecosystems as well as human health [168,169,170]. According to certain research, some plastics, particularly those that contain chemicals like phthalates, can cause a hormone disturbance in animals, which can result in reproductive problems, developmental disorders, and other health issues [171,172,173]. Population dynamics may be impacted by the abilities of these substances to alter sexual differentiation in fish and amphibians [174,175,176,177]. A schematic of every potential environmental health risk factor is presented in Figure 5.

## 5. Possible Risks to Human Health by Airborne MPs/NPs

**Figure 6:**
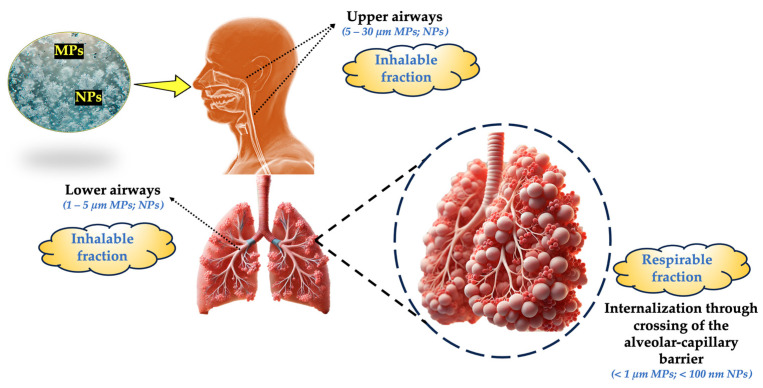
Entry pathways for MPs/NPs via the respiratory system and the site of bioaccumulation.

The ingestion of contaminated food, drink, or even food packaging, as well as the inhalation of dust and air pollution, expose people to airborne MPs and NPs. According to recent studies, MPs/NPs may enter the human body through the air and enter the respiratory system (Figure 6, Table 1), [172,178,179,180].

This is a very important problem because these particles are so small that they can evade the natural defenses of the body and cause respiratory diseases [76,77,181,182]. For instance, MP deposition in the extrathoracic region was the subject of a study. In the tracheobronchial airways and their passage to the digestive system, a thorough examination of the transport behavior and settlement patterns of MPs of different sizes and shapes at varying respiratory intensities was also carried out. Using the MP size distribution found in human lung tissues, the deposition of MPs (1 nm–100 µm) in different areas under varied breathing circumstances showed a greater deposition rate in the nasal cavity. Rapid mucociliary action mainly removed particles that had been accumulated in the thoracic regions: roughly 66% of these particles were moved to the esophagus and finished in about 24 h. The transport rates of 1 mm PE and PTFE particles through the tracheal mucociliary system were 18.5 ± 6.0 and 11.3 ± 3.2 mm/min, respectively. MPs accumulate moderately in the alveolar-interstitial (AI) regions, a highly sensitive deposition site with a thin tissue barrier against particle circulation.

**Figure 7:**
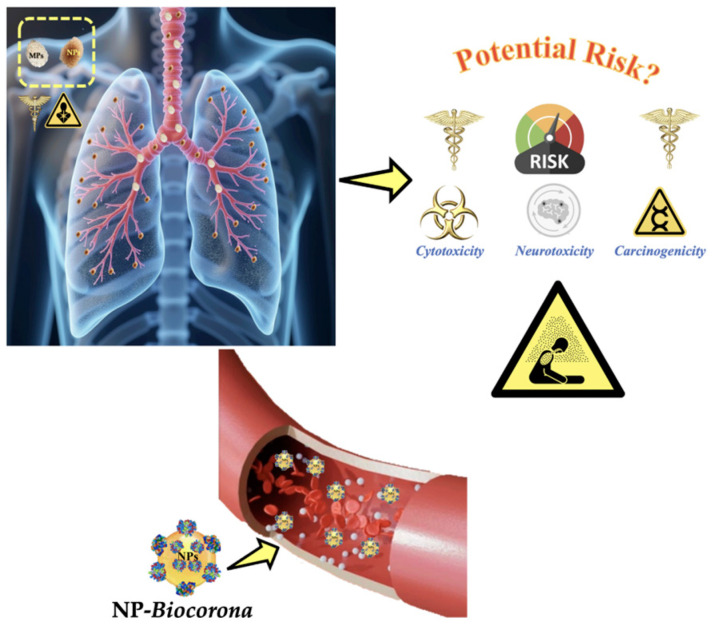
Potential risks to human health caused by MPs/NPs.

Although the potential health effects of breathing in or consuming MPs are still being investigated, there are concerns that they could cause oxidative stress, inflammation, and possibly tissue and cell damage. In particular, NPs may be able to pass through cell membranes due to their small size, which could have more profound biological effects (Figure 7).

Unquestionably, NPs smaller than 10 nm in size have the capacity to penetrate into tissues and enter cells as a gas. Larger NPs and MPs can be absorbed by the airways and by the gastrointestinal system (GI), and human blood contains MPs/NPs [183,184]. After the absorption—whether from airways or the GI—into the cellular compartment, they can interact with proteins, lipids, and nucleic acids to create *biocoronas* on their surface when they migrate through biological fluids. Among the essential physicochemical characteristics that promote the development of *biocoronas* are a high surface/volume ratio, surface charges, and polymer composition [2,185]. Corona constituents are basically either intracellular or extracellular polymeric macromolecules that are produced by the metabolism of the organism. According to the degree of affinity, two layers—soft and hard—are generated as a result of various pressures promoting protein adhesion [2,186].

At the cytotoxicology level, the primary adverse effect of airborne NP release into the inner cell is the disturbance of vital cellular homeostatic functions. Lysosomal damage is a nodal pathological event that happens in a series of stages: (1) immediate damage following the ingestion of NPs in the cells through endocytosis or penetration; (2) attempts by the cell to process these foreign bodies that result in the disruption of autophagy flux and lysosome function; and (3) the breakdown of intracellular material transport between organelles. The oxidation of lipid bilayer constituents such as polyunsaturated phospholipids and glycolipids is dictated by the generation of reactive oxygen species (ROS). As a result, NPs reduce the fluidity and membrane polarization ratio of cell membranes. Additionally, ATP-binding cassette (ABC) transporters, essential membrane proteins that enable the ATP-driven translocation of several substrates across membranes, can interact with NPs [187,188].

At the genotoxic level, when DNA molecules come into contact with PS, PET, PA, and PP polymers, they bond and become unstable. Indeed, chromosomal defects and DNA instability are associated with the translocation of NPs into the nucleus. The phase of the cell cycle determines the carcinogenic effects of NPs. During the interphase, NPs can change transcription (non-genotoxic action) or DNA replication (no genotoxic action) [2] and raise questions regarding the possible long-term effects of chronic exposure, even though additional research is required [2]. The distribution process in target organs such as the lung, brain, and intestine is improved by NPs with reduced diameters. For airborne MPs/NPs’ bioaccumulation and toxicity, the central nervous system (CNS) is one of the main target organs. MPs/NPs can enter the CNS through the vessels upon ingestion, and also the olfactory nerves during inhalation. Both the intracellular concentration of plastic nanomaterial and its deposition affect the susceptibility of the neurons to airborne MPs/NPs. In the brain, the lymphatic system is not present, and once these particles reach the brain they tend to accumulate, because one of the ways to redistribute MPs/NPs is through the lymphatic system [189]. Normal neurotransmission is disrupted by a persistent buildup of airborne MPs/NPs in the neuropil [2,76,181]. The production of ROS, which are extremely reactive molecules created by the interaction with molecular oxygen, is a frequent cause of airborne NP neurotoxicity.

The viability of neural progenitor cells that sustain hippocampus neurogenesis as well as adult neurons is jeopardized by elevated ROS levels [190]. The association between airborne NPs’ exposure and carcinogenesis would be supported by NP-induced anomalies in the cell cycle and resistance to cell death. ROS, oxidative stress (OS) induction, genomic instability, and chronic inflammation all have individual and combined effects that indirectly contribute to NP-induced carcinogenesis. Premature cell death in particular CNS regions brought on by a variety of factors is a hallmark of neurodegenerative diseases. One of the possible causes, according to a body of evidence, may be NP-induced neurotoxicity [2,191].

**Table 1:** An overview of some of the potential hazards that airborne MPs/NPs bring to human health.

Location	MP/NP Type	MP/NP Shape	MPs/NPs Particle Size (µm)	Implications on Human Health	Reference
USA	PA, PE, PET, PP, PS, PVC	Fibers, fragments	0.1–40	Extrathoracic and bronchial regions (upper airways), respiratory tissues	[76]
China	PS	Microbeads	0.50–0.52	Renal injury (NR4A1/CASP3, TF/F12, and HK-2). Renal tubular injury, glomerular mural epithelial cell proliferation, and immune cell infiltration	[77]
China	PE	Microbeads	1.0–5.0	Asthma, higher degree of inflammatory cell infiltration, bronchial goblet cell hyperplasia, oxidative stress injury in the lung (cytokine IL-33 in the BALF)	[181]
China	PS	Microbeads	0.1–1	Liver fibrosis, oxidative stress in AML12 cells, liver inflammation (increasing JNK, JAK1, NF-κB, STAT1, TNF-α, and P38 MAPK)	[182]
China	PHA, PP	Fibers, microbeads	5.0	Intestinal microbiome dysbiosis, intestinal and serum metabolome disruption, hepatic transcriptome disturbances, and hepatotoxicity	[192]
China	PE, PET, PP, PS, PTFE, PVC	Fibers, fragments, films, microbeads	0.5–5.0	Thorax and alveoli, lung tissue, BEAS−2B cells, lung fibrosis damage, COPD, ARDS	[78]
Australia, Bangladesh, USA	PA, PAN, PE, PES, PET, PP, PVC	Fibers, fragments, films, microbeads	0.1–5.0	Hormonal imbalance, undesirable pregnancy outcomes, sexual dysfunction and infertility, asthma, impaired renal function	[193]
Iran	PE, PET, PP, PS	Fibers, fragments, films, microbeads	200–5000	Extrathoracic and bronchial regions (upper airways), respiratory tissues	[20]
China	PAN, PE, PET, PP, PS, PVC	Fibers, fragments, films, microbeads	0.1–5.0	DNA damage, altered gene and protein expression, cell/tissue apoptosis, loss of cell viability, oxidative stress, elevated calcium levels, and inflammation	[163]
China	PS	Microbeads	0.5–3.0	Inflammation in multiple organs, infiltration of neutrophils and macrophages, increased Toll-like receptors (TLRs), myeloid differentiation primary response protein 88 (MyD88) and nuclear factor-κB (NF-κB), as well as proinflammatory cytokines (tumor necrosis factor (TNF)-α and interleukin (IL)-1β) in the lungs, thymus, spleen, liver, and kidneys	[194]
China	PS	Microbeads	0.50–0.52	Glucose metabolism disorder (hyperglycemia), liver damage, liver fibrosis	[195]
China, New Zealand	PS	Microbeads	0.1–5.0	Airway dysbiosis, altered nasal microbiota, altered lung microbiota	[196]
China	EVA, PA, PAN, PE, PET, PMMA, PP, PS, PTFE, PVC	Fibers, fragments, films, microbeads	0.1–500	Extrathoracic and bronchial regions (upper airways), respiratory tissues	[197]

## 6. Future Perspectives and Potential Solutions

The hazard caused by MP/NP pollution is well acknowledged globally; given its direct influence on human health through inhalation, it is remarkable that the atmosphere is the most recent area to be examined in this regard. A possible explanation for this may be that the research procedures that have been created for the study of MPs/NPs are more focused on matrices like soil and water, making them less suitable to air and frequently resulting in incorrect data [198]. The purpose of this review was to demonstrate that there is not a significant correlation between the outcomes of the different sample techniques listed in Tables S1 and S2. Models and comparative chemical/instrumental studies are needed to increase our understanding of the aging, transport, and temporal and spatial dispersion of airborne MPs/NPs [199,200,201,202]. Fibers and fragments, which make up the majority of atmospheric MPs/NPs, are less dense and more gravity-resistant than conventional plastics.

**Figure 8:**
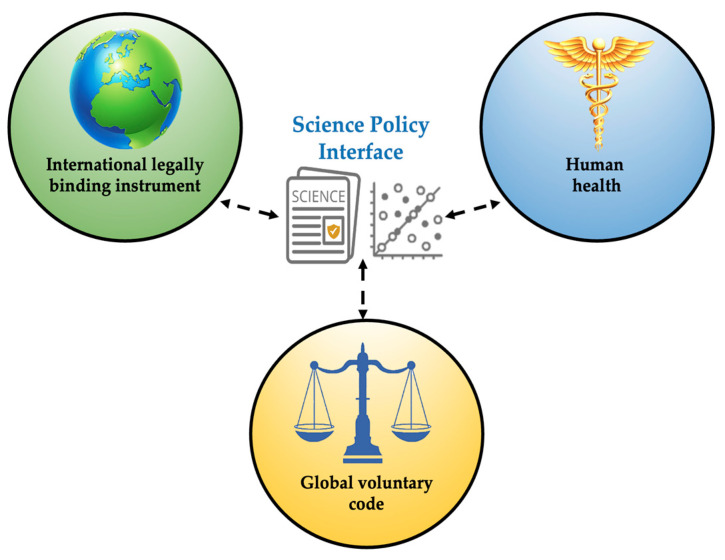
Global Plastic Treaty.

Both individual and industrial modifications are required to lower the quantity of MPs/NPs released into the environment [48]. A dynamic and circular structure including all stakeholders who may be able to offer the necessary solutions must be built to achieve the suggested goals. In order to explore possible solutions, the Scientific Council for Policy of the European Academies (SAPEA) reviewed scientific data gathered on MPs in a 2019 report. One solution, for instance, was implementing doable measures to lessen the likelihood that MPs may leak into the environment. Every place has unique origins and root causes of pollution; therefore, targeted global legislation might address a range of problems [201,203,204]. According to our review, in order to satisfy the evidentiary criteria associated with the Global Plastics Treaty, a better conflict-free science–policy link or connection will be required. Research, state and federal regulations, and community participation are the three pillars of a collaborative society that must work together to solve, or at least minimize, MP generation and its potential adverse effects on the environment and human health. An enhanced and conflict-free science–policy interface pertaining to the Global Plastics Treaty (i.e., airborne MPs/NPs) is shown in Figure 8.

**Figure 9:**
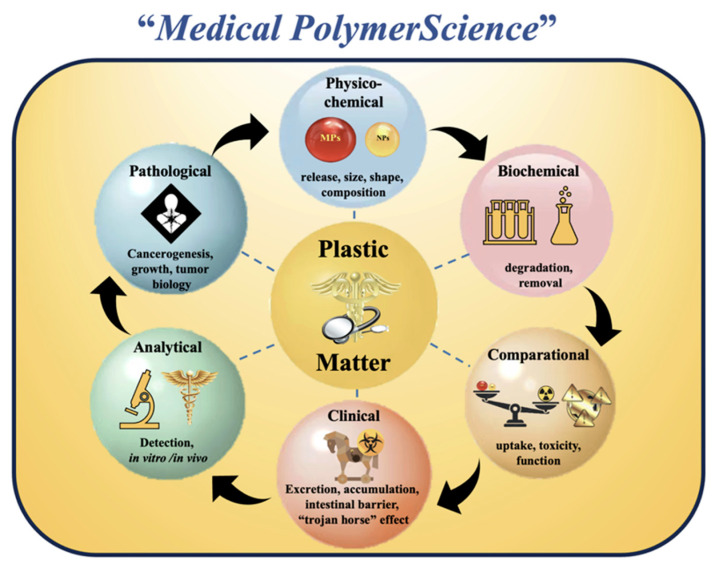
Medical Polymer Science Outline.

New multidisciplinary technologies (communication, social, environmental, health, scientific, etc.) that can promote sustainable development, resource conservation, and environmental sustainability must be put into place. More importantly, though, they must effectively address the issue of plastic materials. Because the presence of MPs/NPs involves a number of scientific branches, it is essential that scientists and other interested parties from a range of scientific disciplines conduct thorough and standardized research that can elucidate the interactions of airborne MPs/NPs with humans and the environment, as well as their potential pathogenic processes [205,206,207]. One example of this new type of interdisciplinary research collaboration is the recently established field of Medical Polymer Science (MPS). This novel scientific approach to MP/NP pollution is diagrammed in Figure 9.

Based on the following criteria, MPS would help to standardize and uniformize the process for identifying airborne MPs/NPs in the environment and human body:

○ Physicochemical characteristics: Dimensions, type of plastic, and shape.
○ Clinical methodology: Amounts of MPs/NPs entering and leaving the body, possible degradation of ingested MPs/NPs’ particles, possible clinical interactions between MPs/NPs and physiological homeostasis and target organs/tissues, permeability/adsorption of MPs/NPs by each target tissue, possible measurement of the *Trojan horse* effect, and studies of signaling pathways.
○ Pathological investigations: Examination of MPs/NPs that initiate, accelerate, and propagate possible carcinogenesis in organs and tissues. For better clinical evaluations, the link between cellular alterations and high MPs/NPs’ concentrations or dosages must be confirmed.
○ Biochemical studies: The possible biochemical processes that contribute to the reduction and destruction of airborne MPs/NPs in tissues and organs must be assessed.
○ Comparative investigation: How MPs/NPs differ from other similar particles that the human body can absorb in terms of absorption, toxicity, and pathological effects (i.e., TiO_2_ nanoparticles) must be examined.

To accurately identify airborne MPs/NPs in complex matrices like the environment and the human body (comprehensive epidemiological investigations), worldwide databases of airborne MPs/NPs must be created using the analyses used in the most recent studies (i.e., Raman spectroscopy, µ-Raman, FTIR, µ-FTIR, and Py-GC/MS).

## 7. Conclusions

Since airborne contaminants have been shown to travel up to 95 km, the detected MPs/NPs show considerable potential for long-range transmission, impacting regions distant from the main sources of pollution [208]. According to all the published studies, there is significant regional variation in the relative abundance of airborne MPs/NPs in terms of both features and concentration levels. The most common types of MPs/NPs found in the atmosphere are fibers and fragments. In particular, synthetic textile fibers are thought to be a major source of MPs/NPs. It is critical to acknowledge the limitations of the existing research, especially in relation to the release and origin of MPs/NPs (i.e., the lack of comparable data on their prevalence and characteristics, particularly in remote locations; non-standardized operating procedures for the sampling and detection methods implemented for MPs/NPs). It should be noted that a notable gap in the literature is the dearth of thorough investigations aimed at identifying the source of atmospheric MPs/NPs.

The current review emphasizes the significance of using consistent MP/NP research methodologies in order to accurately ascertain the prevalence and impacts of these airborne neo-pollutants. To guarantee accurate and comparable results, sampling and analysis methods must be modified to account for the unique constraints posed by various environmental contexts, both urban and remote. Frequent sampling may be necessary to identify periods of heightened pollution, such as peak hours or certain industrial operations, and to detect changes in particulate matter concentrations. The accurate quantification of both big and minute particulate matter is made possible by specialized sample equipment, such as high-capacity air filters, which can handle the high-particle loading typical of metropolitan areas without interference. In surroundings with a high diversity of plastics, advanced chemical analysis techniques like Fourier transform infrared (µFTIR) and Raman spectroscopy (µRaman) are crucial for identifying polymers, particularly certain additions or treatments that are frequently found in urban pollution. Remote locations, on the other hand, provide unique difficulties, since the typically lower MP/NP concentrations are mostly made up of particles that are carried from far-off sources rather than local emissions. In these cases, the detection of MP/NP traces and the understanding of long-range atmospheric transport should be the main goals of standardization techniques.

Since short-term sampling may not provide detectable quantities, long-term monitoring is sometimes required to identify the low but persistent presence of MPs/NPs in remote places. Large amounts of air may need to be analyzed, or other samples may need to be used. In remote areas where MPs may be present at trace levels, analytical methods with low detection limits are essential. Sensitive equipment that can detect particles in the micrometer or even nanometer range is needed. The necessity for flexible yet uniform standards is highlighted by these contextual variations. Biomonitoring can be crucial in tackling one of the most important environmental issues of our day by improving techniques, encouraging interdisciplinary cooperation, and influencing policy choices.

Overall, this review significantly advances our current understanding of airborne MPs/NPs and sets a bar through its thorough methodology, in-depth investigation, and determination of propositional worth. By integrating science, technology, human health, and public policy, it approaches the research of these novel toxins in a unique way.

## Data Availability

No new data were created or analyzed in this study. Data sharing is not applicable to this article.

## Supplementary Materials

The following supporting information can be downloaded at: https://www.mdpi.com/article/10.3390/toxics13050387/s1. Table S1: Studies published in the literature on outdoor MPs/NPs contamination; Table S2: Studies published in the literature on indoor MPs/NPs contamination. References [209,210,211,212,213,214,215,216,217,218,219,220,221,222,223,224,225,226,227,228,229,230,231,232,233,234,235,236,237,238,239,240,241,242,243,244,245,246,247,248,249,250,251,252,253,254,255,256,257,258,259,260,261,262,263,264,265] are cited in the supplementary materials.

## Abbreviations

The following abbreviations are used in this manuscript:

ABC	ATP-binding cassette
ABS	Acrylonitrile butadiene styrene
AFM-IR	Atomic force microscopy-based infrared
AI	Alveolar-interstitial regions
ARDSS	Acute respiratory distress syndrome
ASA	Acrylonitrile styrene acrylate
ATR-FTIR	Attenuated total reflectance–Fourier transform infrared spectroscopy
BPA	Bisphenol A
CNS	Central nervous system
COPD	Chronic obstructive pulmonary diseases
EPDM	Polyethylenepropylene-diene
EVA	Ethylene vinyl acetate
FPA	Focal plane array
FSEI	Fusion and solvent evaporation ionization
FTIR	Fourier transform infrared spectroscopy
GHG	Greenhouse gas
GI	Gastrointestinal system
HDPE	High-density polyethylene
LDIR	Laser-direct infrared
LDPE	Low-density polyethylene
LIBS	Laser-induced breakdown spectroscopy
MO	Microorganisms
MPs	Microplastics
MPS	Medical Polymer Science
NMR	Nuclear magnetic resonance
NEE	Non-exhaust emission
NPs	Nanoplastics
NY	Nylon
OS	Oxidative stress
PA	Polyamide
PA-6,6	Polyamide 6,6
PFAS	Perfluoroalkyl and polyfluoroalkyl substances
PFOA	Perfluorooctanoic acid
PAH	Polycyclic aromatic hydrocarbon
PAI-TOFMS	Photoinduced associative ionization time-of-flight mass spectrometry
PAN	Polyacrylonitrile
PAN/PS/PMA	Poly(acrylonitrile:styrene:methyl acrylate)
PB	Polybutylene
PBA	Poly(11-bromoundecyl acrylate)
PBD	Polybutadiene
PBDE	Polybrominated diphenyl ethers
PC	Polycarbonate
PCB	Polychlorinated biphenyl
PCL	Polycaprolactone
PDMS	Polydimethyl siloxane
PE	Polyethylene
PTFE	Polytetrafluoroethylene (Teflon)
PES	Polyester
PET	Polyethylene terephthalate
PHA	Polyhydroxyalkanoates
PHB	Polyhydroxy butyrate
PLA	Polylactic acid
PLE	Pressurized liquid extraction
PM	Particulate matter
PMMA	Polymethylmethacrylate
POM	Polyoxymethylene
POPs	Persistent organic pollutants
PP	Polypropylene
PS	Polystyrene
PVA	Polyvinyl alcohol
PVC	Polyvinyl chloride
PU	Polyurethane
PUPA	Polyurethane and poly(amido-amine)
Py-GC/MS	Pyrolisis GC/MS spectrometry
RA	Rayon
ROS	Reactive oxygen species
SAN	Styrene acrylonitrile
SBR	Styrene-butadiene
SEM	Scanning electron microscopy
SERS	Surface-enhanced Raman spectroscopy
SPR	Surface plasmon resonance
TWPs	Tire wear particles
WWTPs	Wastewater treatment plants
µFTIR	Micro Fourier transform infrared spectroscopy
