# Supplementary material for: Plastic Smell: A Review of the Hidden Threat of Airborne Micro and Nanoplastics to Human Health and the Environment
Source: Toxics. 2025 May 12;13(5):387. doi: 10.3390/toxics13050387 (PMC12115425; doi:10.3390/toxics13050387)
Supplement: Supplementary file 1 [file toxics-13-00387-s001.zip › toxics-3592383-supplementary.pdf]

Supporting Information

Plastic Smell: A Review of the Hidden Threat of Airborne Micro and Nanoplastics to Human Health and the Environment

Claudio Casella Ph.D. [1], Dr. Umberto Cornelli MD [2], Santiago Ballaz Ph.D. [3], Giuseppe Zanoni Ph.D. [1], Gabriele Merlo Ph.D. [1], and Luis Ramos-Guerrero Ph.D. [4*]

[1]Department of Chemistry, University of Pavia, Viale Taramelli 12, 27100 Pavia, Italy

[2]School of Medicine, Loyola University, Chicago, IL 60660, USA

[3]Faculty of Health Sciences, Universidad del Espiritu Santo, Samborondón P.O. Box 09-01-952, Ecuador

[4]Grupo de Investigación en Bio-Quimioinformática, Carrera de Ingeniería Agroindustrial, Facultad de Ingeniería y Ciencias Aplicadas, Universidad de Las Américas (UDLA), Quito 170513, Ecuador

Table S1. Studies published in the literature on outdoor MPs/NPs contamination

Location	Sample Type	MP/NP concentration	Polymer Type	Shape	Size (μm)	Colour	Analytical Technique	Reference
University of Hull, United Kingdom	Outdoor airborne	-	EPDM, PA, LDPE, PS, PTFE	Fragments, fibres	10 - 750	Black, blue, red, green, grey, transparent, white	μFTIR spectroscopy	[64]
China	Outdoor air	-	PE, PET, PP, PS, PVC	-	-	-	PCA	[119]
Luján, Argentina	Urban outdoor air deposition	12- 20 MPs/g	HDPE, PA, PES, PP, PU, PVC	Fibres, fragments	900 - 1,553	-	Stereoscopic microscope, hot needle test	[209]
Mid-Southwest coast, Taiwan	Outdoor air	1.89 ± 1.80 particles/m^3	LDPE, PA, PBT, PET, PMMA, PP, PTFE, PVA	Fibres, fragments, films, microbeads	3 - 5,000	-	Stereoscopic microscope, μRaman spectroscopy	[210]
Qilian Mountains, China	Outdoor dust air	4.07 ± 2.37 particles/m^3	PA, PE, PET, PMMA, PP, PS, PTFE, PU, PVA, PVC	Fibres (11%), fragments (89%)	50	-	LDIR	[26]
Thulamela Local-Municipality, South Africa	Urban, rural, forest atmospheric deposition	355.6 ± 47.6 particles/m^2/day (urban area); 189.5 ± 20.0 particles/m^2/day (rural area); 90.5 ± 15.2 particles/m^2/day (forest area)	PET (42.3%), PE (27.2%), PP (17.2%), PS (10.6%), PVC (1.2%), PA (0.9%), PVA (0.6%)	Fibres (89.2%), film (8.7%), foam (1.6%), fragments (0.5%)	100 - 1,000	Black, blue, green, red, transparent, while, yellow	Dissecting microscope, FTIR spectroscopy	[66]
Southern coast of Aotearoa, New Zealand	Aerosol atmospheric deposition	4,885 ± 1,858 particles/m^2/day	PE, PET, PMMA, PP, PS, PVC	Fibres, fragments	1 - 200	-	Fluorescence microscopy, Pyr-GC/MS	[67]
Incineration power plant, China	Flue gas	12 · 10^{12} particles/year	PAM, PVC	Fibres, fragments	10 - 40	-	Kevley microscope, LDR, SEM-EDX, μRaman spectroscopy	[211]
Kumamoto City, Japan	Road Dust	57,500 – 160,000 particles/kg dw	PET, PMMA	-	100 - 1,000	-	FTIR spectroscopy	[212]
Tokio, Japan	Urban road dust	18 ± 4.3 particles/g	CB, CR, CSM, EPDM, HNBR, NBR, PU	-	-	Black, orange	Stereoscopic microscope, ATR-FTIR	[71]
Taichung City, Taiwan	Outdoor air (House)	10.9 ± 14.1 particles/m^3	PA, PAA, PEO, PET, PLA, PTFE	Fragments (62%), film (26%), f ibres (7%), microbeads (5%)	3 - 5,000	-	Microscope, μRaman spectroscopy	[72]
Sarakhs, northeast Iran	Dust storm	0.48 - 23.0 MPs/g	HDPE, LDPE, PA, PET, PP, PS, PTFE, PVC	Fibres, fragments, films	100 - 1,000	-	Binocular microscope, μRaman spectroscopy	[213]

Table S1. *Continued*

Hull and Bristol, United Kingdom	Urban rooftop, outdoor rooftop	1,455 – 2,328 particles/m²/day	EVA, PBMA, PE, PMA, PP, PS, PTFE	Fibres	101.8 – 120.4	-	μFTIR spectroscopy	[132]
South Appalachia, USA	Outdoor air	68 MPs/m²/d	PA, PE, PET, PS, PTFE	Fibres (51%), fragments (40%), films (9%)	39 – 3,743	-	Fluorescence microscope, stereomicroscope, μRaman spectroscopy	[214]
Southeastern Brazil, Brazil	Outdoor air in elementary school	60 items/m²/d – 168 items/m²/d	EVA, PE, PES, PET	Fibres, fragments, microbeads	10 – 5,000	Black, blue, green, red, white	Optical microscopy, μRaman spectroscopy	[215]
Iligan City, Phillipines	Road Dust	0.5 MPs/g	EPR, EVA, PAM, PES	Fibres	10 – 5,000	Blue	Microscope, FTIR analysis	[216]
Chima	Urban outdoor air deposition	512 items/m²/d	PA, PE, PET, PP	Fibres, fragments, pellets	20 - 2,302	-	LDIR	[217]
Ahvaz, Iran	Industrial and urban outdoor air deposition	0 - 1.7 · 10^{-2} particles/m³	PA, PET, PP	Fibres	100 - 1,000	-	Binocular microscopy, SEM/EDX, μRaman spectroscopy	[218]
Seoul, South Korea	Suspended	0.33 - 1.21 items/m³	PA, PET, PP, PS, PU	Fragments (87%), Fibres (13%)	148 - 2,278	-	μFTIR microscopy	[219]
Multiple locations, Spain	Urban outdoor air deposition	5 – 79 MPs/m²/d	ACR, PA, PE, PES, PP, PS, PVC	Fibres (70%), fragments (29%), films (1%)	16 – 1,014	Black, blue, red, transparent, white, yellow,	Stereomicroscope, μFTIR microscopy	[220]
Malaysia	Depositions	114 – 689 items/m³	PA, PA-6,6, PE, PES, PP, PVC	Fragments, Fibres	5 – 5,000	-	Digital camera coupled to a macro lens, μFTIR microscopy	[221]
São Paolo, Brazil	Urban outdoor air deposition	123 - 309 particles/m²/day	PP, PE, PES, PET	Fragments, films, foams, granules	50 - 2,726	-	Fluorescence microscope, FTIR	[222]
São Paolo, Brazil	Depositions	23 - 47 particles/m²/day	PAN, PE, PES, PVC	Fragments (74%), film (13%), granules (8%), foams (5%)	50 - 877	-	Fluorescence microscope, ATR-FTIR	[51]
Humber region, United Kingdom	Urban outdoor air deposition	3,055 - 5,072 particles/m²/day	PA, PE	Fragments, films	10 - 3,000	-	μFTIR microscopy	[223]
Poland coast, Baltic Sea and Gotland Island	Suspended	0 - 682 particles/m³	PE, PE, PES, PET, PUPA	Fibres (98%), fragments (2%)	14 - 509	Blue, green, purple, red, transparent	μRaman spectroscopy	[224]

Table S1. *Continued*

Lanzhou, China	Fallout	354 - 1,159 particles/m²/day	PA, PE, PET, PMMA, PS, PVC	Fibres (48%), fragments (44%)	50; > 1,000	Black, blue, transparent	Stereomicroscope, Raman spectroscopy	[38]
Xi'an, Northwest China	Outdoor air	0.8 - 12.5 particles/L	PE, PET, PP, PS, PVC	Fragments, films, foams, granules	10 - 1,000	-	Stereomicroscope, Raman spectroscopy	[57]
Sri Lanka	Outdoor air	0 - 0.9 particles/m³	PES, PET	Fibres, fragments	50 - 5,000	-	Stereomicroscope, FTIR	[225]
Jakarta, Indonesia	Fallout	3 - 40 items/m²/day	PB, PE, PET, PS	Fibres, fragments, foams	300 - 1,000	-	Stereomicroscope, FTIR	[226]
Mexico City, Mexico	Urban outdoor wet and dry deposition	0.11 - 021 particles/m³	PA, PE, PET, RA	Fibres	39 - 5,000	-	Microscope with hot needle, ATR-FTIR	[35]
Western Pacific Ocean	Oceanic air	0.70 - 0.84 particles/100 m³	PA, PET	Fibres	35 - 1,379	Black, blue, green, red, transparent, yellow	Stereomicroscope, µFTIR	[227]
Ontario, Canada	Urban outdoor air deposition	4 - 9 particles/m²/day	PA, PET	Fibres (89%), fragments (11%)	20 - 4,980	Blue, green, red	Stereomicroscope, µRaman spectroscopy	[129]
Persian Gulf, Iran	Urban outdoor air deposition	14.2 particles/m³	PA, PE, PET, PP, PS	Fibres, fragments, films	2.5 - 10	Black, grey, orange, red, transparent	Binocular microscope, µRaman spectroscopy	[228]
Pic du Midi observatory, France	Suspended	0.09 - 0.66 particles/m³	PE, PET, PP, PS, PVC	Fragments, fibres	10 - 53	-	Stereomicroscope, µFTIR	[229]
Nam Co Basin, Tibet, China	Remote outdoor air deposition	500 kg/year	PA, PET, PP	Fibres, fragments, films, foams, microbeads	50 - 5,000	Blue, green, grey, purple, red, white, yellow	Stereomicroscope, µFTIR	[230]
Madrid, Spain	Atmospheric boundary layer, Suspended	1.5 - 14 particles/m³	PA, PB, PE, PES, PP, PU	Fragments, fibres	10 - 500	-	Stereomicroscope, µFTIR	[231]
Guangzhou, China	Urban outdoor wet and dry deposition	51 - 178 particles/m²/day	PE, PET	Fibres, fragments, films, microbeads	50 - 5,000	-	Stereomicroscope, µFTIR	[232]
Wenzhou, China	Suspended	101 - 224 particles/m³	PE, PES, PS	Fragments (96%)	5 - 5,000	Black, blue, grey	Stereomicroscope, µFTIR	[233]
Brisbane, Australia	Urban road dust	0.7 - 5.9 mg/g	PE, PET, PMMA, PP, PS, PVC	Fragments	250 - 5,000	-	Pyr-GC/MS analysis	[110]

Table S1. *Continued*

Ho Chi Minh City, Vietnam	Urban outdoor wet and dry deposition	71 - 917 items/m²/day	PE, PP, PVC	Fibres, fragments	300 - 5,000	-	ATR-FTIR	[234]
Northern cities of China	Urban outdoor air	358 ± 132 items/m³	PA, PE, PET, PP, PS, PVC	Fibres, fragments	5 - 300	-	Stereomicroscope, µFTIR	[235]
French Atlantic, France	Seaspray	0.05 - 0.06 MP/m³	PET, PP, PS, PVC	Films, fibres	8 - 140	-	Stereomicroscope, µRaman spectroscopy	[5]
Multiple protected areas, USA	Fallout	132 items/m²/day	PA, PE, PES, PP, PVA	Fibres	4 - 188	-	Stereomicroscope, µFTIR	[236]
London, United Kingdom	Outdoor air	21 - 236 items/m³	PA, PE, PET, PP, PS, PVC.	Fibres, fragments	2 - 10	-	Stereomicroscope, µRaman spectroscopy	[23]
Beijing, China	Urban outdoor air	16.7 · 10⁻³ fibers/mL	-	Fibres	-	-	SEM-EDX	[237]
Multiple locations, Ireland	Coastal outdoor wet deposition	80 microfibres/m²/day	PAN, PE, PET, PP	Fibres	40 - 19,750	-	Stereomicroscope, Raman spectroscopy	[238]
Israel	Remote marine atmosphere	-	PE, PP, PS	-	5 - 500	-	Stereomicroscope, µRaman spectroscopy	[239]
South China Sea and East Indian Ocean	Suspended atmospheric particles	0.4 ± 0.6 items/100 m³ – 4.2 ± 2.5 items/100 m³	PA, PAN, PE, PET, PP	Fibres (80%), fragments (20%)	288 – 1861	Black, blue, brown, red, white, yellow	Stereomicroscope, µFTIR	[162]
London, United Kingdom	Urban outdoor air deposition	771 particles/m²/day	PAN, PA, PE, PES, PET, PP, PS, PVC, PU	Fragments, films, granules, foams	75 – 1,080	-	Fluorescence stereomicroscope, µFTIR	[45]
Asaluyeh County, Iran	Urban dust	0.3 -1.1 particles/m³	-	Fibres, films, microbeads, fragments	25 – 2,600	Blue, red, transparent, white, yellow	Binocular microscopy, SEM/EDS	[96]
French Pyrenees, France	Remote outdoor wet and dry deposition	296 – 455 items/m²/day	PE, PET, PP, PS	Fragments (68%), fibres, films	25 - 5,000	Black, blue, green, orange, purple, white	Stereomicroscope, µFTIR	[58]
Hamburg, Germany	Urban and rural outdoor air deposition	136 - 512 items/m²/day	EVA, PE, PET, PTFE, PVA	Fragments (95%), fibres (5%)	63 - 5,000	-	Fluorescence microscopy, µRaman spectroscopy	[240]
Shangai, China	Suspended outdoor dust	0 - 4 items/m³	EVA, PAN, PE, PES, PET, RA	Fibres (67%), fragments (30%), granules (3%)	23 - 9,955	Black, red, transparent	Stereomicroscope, µFTIR	[241, 242]
Shangai, China	Suspended outdoor dust	1,4 - 2,8 particles/m³	EVA, PAA, PAN, PE, PES, PET, RA	Fibres, fragments, granules	23 - 5,000	Black, blue, brown, green, grey, red, transparent, yellow	Stereomicroscope, µFTIR	[243]

Table S1. *Continued*

Nottingham, United Kingdom	Urban outdoor air deposition	3 - 128 fibres/m²/day	-	Fibres	-	-	Stereomicroscope, μFTIR	[244]
Surabaya, Indonesia	Urban outdoor air deposition	130 - 175 particles/m³	PET, PES, PP	Fibres (98%), fragments (1%), films (1%)	1,000 - 1,500	-	Digital microscope, FTIR	[245]
Sakarya, Türkiye	Suspended outdoor air	9,067 - 30,800 particles/m³	PA, PE, PES, PP, PU	Fibres, fragments	50 - 500	-	Stereomicroscope, μFTIR	[246]
Bushehr City, Iran	Street dust	210 - 1,658 · 10^{-3} mg/g	PA, PET, PP	Fibres, fragments	2.5 - 10	-	Fluorescence microscopy, SEM–EDS	[247]
Dongguan, China	Urban outdoor air deposition	175 - 313 particle/m²	PE, PP, PS	Fibres, foams, fragments, films	220 - 4,200	Black, blue, red, white, yellow	Fluorescence microscopy, SEM–EDS	[105]
Paris, France	Urban outdoor dust deposition	0.3 - 59 particles/m³	PA, PE, PP	Fibres	50 - 3250	-	Stereomicroscope, μFTIR	[97]
Yantai, China	Urban outdoor air deposition	400 particles/m²/day	PE, PET, PS, PVC	Fibres, fragments, films, foams	50 - 1,000	Black, red, transparent, white	Stereomicroscope, μFTIR	[248]
Paris, France	Urban outdoor air deposition	110 - 210 items/m²/day (urban); 53 - 92 items/m²/day (sub-urban)	-	Fibres	50 - 5,000	-	Stereomicroscope, μFTIR	[6]
Paris, France	Urban outdoor air deposition	29 - 280 particles/m²/day	-	Fibres (90%), fragments (10%)	100 - 5,000	-	Stereomicroscope, μFTIR	[53]

Table S2. Studies published in the literature on indoor MPs/NPs contamination

Location	Sample Type	MP/NP Concentration	Polymer Type	Shape	Size (μm)	Colour	Analytical technique	Reference
Japan	Indoor house dust	50 particles/household	CE, PA, PAN, PE, PET	Fibres (90%), fragments, foams, pellets, films	45 - 2,000	-	SEM, μFTIR	[74]
China	Exhaled breath biomarkers associated with MP exposure	22.5 mg/kg MPs in 10 μL saline for 30 days	PS	Microbeads	0.1 - 10	-	PAI-TOFMS	[249]
Malaysia	Indoor air	599 ± 182 in offices, 399 ± 52 in classrooms, 505.17 ± 203.78 in apartments, and 515 ±134 in landed homes	PA, PAN, PC, PMMA	Fibres, fragments, foams, films, pellets	170 - 1,000	Black, blue, orange, red, transparent	Stereomicroscope, Raman spectroscopy	[75]
Taichung City, Taiwan	Indoor air (House)	8.0 ± 10.7 particles/m^3	PA, PAA, PEO, PET, PLA, PTFE	Fragments (50%), films (33%), fibres (13%), microbeads (4%)	3 - 5,000	-	Microscope, μRaman spectroscopy	[72]
China	Exhaled breath biomarkers associated with MP exposure	0.75 - 5 mg/kg_{bw} for 42 days	PHA, PP	Microbeads	5	-	Stereomicroscope	[192]
Birmingham, United Kingdom	Indoor air	[illegible]60 MP/m^2/day (workplace); 3,[illegible] MP/m^2/day (homes) day	PA, PE, PES, PET, PP, PVC	Fibres (75%), fragments (22%), foams (3%) for homes; fibres (70%), fragments (21%), foams (9%) for workplaces	10 - > 100	-	Fluorescence microscope, μFTIR	[80]
Türkiye	Indoor air	9 - 14 MPs/house	HDPE, LDPE, PA 6, PE, PMMA, PP, PVC	Fibres, fragments, foams, pellets, films	179 - 3,714	Black, blue, brown, green, red, transparent, yellow	Optical microscope, μRaman spectroscopy	[14]
Aarhus, Denmark	Indoor air (laboratory)	$4 \cdot 10^7$ - $2 \cdot 10^9$ particles/cm^3	PS	Microbeads	0.10 - 0.27		Pyr-GC/MS analysis	[85]
Ahvaz, Iran	Indoor air	48 MPs/m^3	PE, PET, PP, PS, PVC	Fibres (20%), fragments (7%), films (4%), microbeads (69%)	20 - 5,000	Black, blue, grey, orange, transparent, white, yellow	PCA	[20]

Table S2. *Continued*

Iran	Classroom	81 - 55,830 MPs/g	PA, PET, PP, PS	Fibres	< 100; > 1,000	Black, blue, green, grey, red, transparent, white, yellow	Binocular microscope, SEM-EDX	[46]
Pakistan	House	29 - 636 fibres/m^2	PE, PET, PP, PTFE	Films, fragments, fibres, foams, nurdles	-	Blue, black, grey, orange, red, transparent, white, yellow	Fluorescence microscope, μFTIR	[250]
Jiangsu, China	Indoor fallout	78,839 - 96,367 MPs/m^2/day	-	Fragments (89%)	20 - 300	-	LDIR, μFTIR	[251]
Wenzhou, China	Indoor deposition	$7.6 \cdot 10^5$ items/m^2/day	PA, PE, PES, PET	Fragments (88%)	6 - 1,000	-	Fluorescence stereomicroscope, μFTIR	[84]
Iran	Hospital, mosque, kindergarten, university, house	49 – 139 items/mg	PA, PC, PE, PET, PP	Fibres, films, fragments	< 100; > 1,000	Black, blue, green, red, white, yellow	Binocular microscope, μRaman spectroscopy	[252]
Iran	School	10 - 635 MPs/g	PA, PET, PP, PS	Fibres, fragments, sheets	50 - 1,000	Black, blue, red, transparent, white, yellow	Binocular microscope, SEM-EDX	[253]
Yangling, China	Indoor fallout	1,404 - 5,844 MPs/m^2/day	PA, PAN, PET, PP, PVC	Fibres, films	< 500; 5,000	Black, blue, green, red, white	Metallographic microscope, ATR-FTIR, μFTIR	[82]
Netherlands	Indoor air (House)	0.31 – 305 mg/g	PET	-	-	-	LC-UV, LC-ESI-MS	[254]
Barcelona, Spain	Indoor air	4.2 – 17.3 MPs/m^3	PA, PES, PP	Fibres, fragments	20 – 23,565	Blue, black, grey, purple, red, transparent	Stereomicroscope, μFTIR	[255]
Shanghai, China	Indoor air	15.6 – 93.3 items/m^3	PE, PES, PP, PU, PVC	Fragments (85%), films, microbeads	2.4 – 2,182	Black, blue, green, indigo, p8ink, purple, red	Stereomicroscope, Raman spectroscopy	[256]
New Jersey, USA	Indoor deposition	$4.5 \cdot 10^3$ - $1.09 \cdot 10^4$ items/m^2/day	-	Fibres, films	-	-	Binocular microscope, SEM-EDS, Raman spectroscopy	[16]
China	Apartment, classroom, dormitory, hotel, office	62 - 3,861 MPs/g	PA, PE, PET, PC, PP, PVC	Fibres, films, fragments	200 - 1,000	Black, green, red, transparent, white	Stereomicroscope, μFTIR	[257]
Humber region, United Kingdom	Indoor deposition	0 - 5,412 items/m^2/day	PA, PAN, PE, PET, PMMA, PP	Fibres (90%), fragments (8%), films (2%)	5 - 5,000	-	Stereomicroscope, μFTIR	[223]

Table S2. *Continued*

Wenzhou, China	Indoor air	1,181 - 1,583 items/m^3	PA, PES, PP	Fragments (90%)	5 - 5,000	-	Fluorescence stereomicroscope, μFTIR	[233]
Queensland, Australia	Urban road dust	1.6 - 1.8 fibres/m^3	PET	Fibres	19 - 3,948	Blue	Pyr-GC/MS analysis	[110]
Sydney, Australia	Indoor deposition	22 - 6,169 items/m^2/day	PA, PE, PES, PET	Fibres (99%)	50 - 5,000	Blue, black, brown, green, grey, transparent	Stereomicroscope, μFTIR	[258]
Aveiro, Portugal	Indoor air	1.1 - 3.9 items/m^3	-	Fibres, fragments, microbeads	9.6 - 10,822	Black, bliue, brown, purple, white, yellow	Optical microscope, stereomicroscope	[259]
California, USA	Air	3.3 fibres/m^3; 12.6 fragments/m^3	PE, PET, PS, PVC	Fibres, fragments	55 - 58.6	-	Stereomicroscope, μFTIR, μRaman spectroscopy	[98]
China	Indoor deposition	23.04 items/m^2/d - 67.54 items/m^2/d	-	-	100 - 397	-	Stereomicroscope, μFTIR	[153]
China	Dormitory	18 - 43 mg/kg PA 6; 54 - 321 mg/kg PA66	PA 6, PA 66	-	-	-	Stereomicroscope, μFTIR	[260]
Shanghai, China	Deposition	500 - 29,000 items/m^2/day	PA, PE, PP, PS, RA	Fibres	50 - 2,000	Black, blue, green, purple, red, transparent, yellow	Pyr-GC/MS analysis	[261]
12 Countries	Bedroom, living room	38 - 120,000 mg/kg PET; 0.11 - 1,700 mg/kg PC	PC, PET	-	-	-	Stereomicroscope, μFTIR	[262]
China	Home	1,550 - 120,000 mg/kg PET; 1 - 100 mg/kg PC	PC, PET	Fibres, granules	50 - 2,000	-	ESI–MS/MS	[263]
Aarhus, Denmark	Air	1.7 - 16.2 items/m^3	PAN, PE, PES PP	Fragments (87%), fibres (13%)	4 - 398	-	FPA-μFTIR-Imaging spectroscopy	[264]
Edinburgh, United Kingdom	Deposition	1,667 - 1,672 items/m^2/day	PET, PU	Fibres	≤ 500	-	Stereomicroscope, μFTIR	[265]
Paris, France	Indoor air (Apartment, office); deposition	0.4 - 59.4 fibres/m^3 (indoor air); 1,600 - 11,000 fibres/m^3/day	PA, PE, PP, PVC	Fibres	50 - 4,850	-	Stereomicroscope, μFTIR	[97]
